# Performance of dental students, orthodontic residents, and orthodontists for classification of midpalatal suture maturation stages on cone-beam computed tomography scans – a preliminary study

**DOI:** 10.1186/s12903-024-04163-3

**Published:** 2024-03-22

**Authors:** Sachin Chhatwani, Annahita Arman, Stephan Christian Möhlhenrich, Björn Ludwig, Jochen Jackowski, Gholamreza Danesh

**Affiliations:** 1 Department of Orthodontics, School of Dentistry, Faculty of Health, Witten/Herdecke University, Alfred-Herrhausen Str. 50, Witten, 58448 Germany; 2 Private Practice, Kleve, Germany; 3 Department of Orthodontics, University of Saarland, Campus Homburg, Homburg/Saar, Germany; 4 Department of Oral Surgery and Dental Emergency Care, School of Dentistry, Faculty of Health, Witten/Herdecke University, Alfred-Herrhausen Str. 50, Witten, 58448 Germany

**Keywords:** Midpalatal suture maturation, Palatal expansion, Cbct, Orthodontics

## Abstract

**Background:**

Assessment of midpalatal suture maturation on cone-beam computed tomography (CBCT) scans is performed by visual inspection and is therefore subjective. The extent to which the assessment of midpalatal suture maturation is affected by rater experience has not been adequately explored in the existing literature, thus limiting the availability of evidence-based findings. This study compared the outcomes of classification by dental students, orthodontic residents, and orthodontists.

**Methods:**

Three different groups of students, orthodontic residents, and orthodontists evaluated 10 randomly chosen CBCT scans regarding midpalatal suture maturation from a pool of 179 patients (98 female and 81 male patients) aged 8 – 40 years which were previously classified by evaluating CBCT scans. The pool was set as benchmark utilizing midpalatal suture maturation classification by one examiner (OsiriX Lite version 11.0; Pixmeo SARL, Bernex, Switzerland). For assessment of intra-rater reliability of the examiners of each group the randomly chosen subjects were reclassified for midpalatal suture maturation after a wash-out period of two weeks by using the same software. Statistical analysis was performed to evaluate intra- and interrater reliability of the three groups with differing experience level.

**Results:**

Groupwise intra-rater reliability assessment between the classification and reclassification was weak for examiners with a low level of experience (k = 0.59). Orthodontists had highest degree of agreement with regard to benchmark classification with an inter-rater reliability to be considered as moderate (k = 0.68).

**Conclusions:**

Assessment of midpalatal suture maturation on CBCT scans appears to be a subjective process and is considerably related to the experience level of the examiner. A high level of clinical experience seems to be favorable but does not necessarily ensure accurate results.

## Background

Rapid maxillary expansion (RME) is a common orthodontic procedure used to treat transverse discrepancies. Although surgically assisted RME, also known as surgically assisted rapid palatal expansion (SARPE), is more frequently employed in adults, conventional RME is typically performed in children and adolescents [1]. The need of SARPE is determined on the basis of distinct features. Skeletal involvement is indicated by the presence of more than two teeth in a crossbite [2], and a presence of at least 5% ossification of the midpalatal suture indicates the need for SARPE since it can increase the transverse osseous resistance to conventional expansion [3]. The findings of finite element analysis indicate that the material properties of the midpalatal suture and the circummaxillary sutures have a significant impact on the pattern of expansion [4], this indicates that the characteristics of the sutures play a significant role in maxillary expansion.

On the basis of a visual examination of cone-beam computed tomography (CBCT) results, Angelieri et al. classified the sutural appearance of the midpalatal suture into five maturation stages (A–E). Angelieri et. state that the stages A and B were not anticipated to exhibit greater bone resistance, and when stages D and E were identified, surgically assisted therapy was recommended [5]. However the recommendations made were based on qualitative assessment of the midpalatal suture, without performing and measuring outcome of surgical intervention or consideration of ossification as a measured parameter.

The reliability of diagnostic evaluations is crucial to minimize errors and ensure consistent outcomes, regardless of variables such as the examination environment, the time of assessment, and the examiner [6]. Reliable means of assessing midpalatal suture maturity could enhance the selection of optimal treatment strategies for the patient [7]. As the classification approach proposed by Angelieri et al. employs CBCTs, which result in a higher radiation dose, it is crucial to ensure the reliability of the assessment. This is particularly significant since the classification using CBCT has been suggested for patients aged between 14 and 18 years [8].

Thus, although classification based on the criteria by Angelieri et al. showed strong to moderate agreement for intra-examiner and inter-examiner agreement [9], the ratings themselves may be affected by the experience level of the rater. It has been shown that classification of midpalatal suture maturation performed by visual inspection by humans, is not free of subjectivity and requires a high level of technical sensitivity [10]. This is supported by another investigative study on the reliability of midpalatal suture maturation evaluation via CBCT imaging, which found the inter-examiner reliability to be moderate to weak, and thereby advise that the application of this approach should be considered with caution [11]. The assessment of midpalatal suture maturation necessitates extensive training for the examiner [12].

The aim of this study was therefore to evaluate the influence of raters’ experience on the classification of midpalatal suture maturation. For this we compared the performance for midpalatal suture maturation stage assessment, as described by Angelieri et al., of dental students, orthodontic residents, and orthodontists.

The null hypothesis was that the assessed maturation stage of the midpalatal suture would show no differences among the groups.

## Methods

Ethical approval for this observational comparative study, which utilized retrospectively analyzed data, was provided by the Ethics Committee of the University of Witten/Herdecke (approval no. 291/2021). The original pool of CBCT images was obtained from 547 patients who were treated at the Dental Clinic of the University of Witten/Herdecke, Germany in the years 2015–2016. Sample size determination was not undertaken for this preliminary study.

All CBCT images were generated in Digital Imaging and Communications in Medicine (DICOM) format with GALILEOS Comfort (Sirona Dental Systems GmbH, Germany) at an X-ray exposure of 85 kV and 5–7 mA (14 s; field of view: 150 × 150 mm, 200 singular images), yielding a voxel size of 0.027 mm^3^ and slice thickness of 300 µm.

The inclusion criteria for this study were as follows: patients aged between 8 and 40 years who had not received prior orthodontic or surgical treatment and had a CBCT image of adequate quality.

Patients with a history of craniofacial anomalies such as cleft lip and palate, cysts or tumors in the maxillary region, and CBCT scans with subjectively insufficient image quality were excluded.

The patient data were pseudonymized at the source. All CBCT scans were analyzed using OsiriX Lite version 11.0 (Pixmeo SARL, Bernex, Switzerland) in a dark room with an X-ray reporting monitor under the same screen settings. The 179 CBCT images were initially assessed and classified by a single examiner, a dentist who had received training in CBCT diagnosis. This examiner utilized dynamic free scanning to classify the images based on the classification system developed by Angelieri et al. [5]. We refer to the existing literature for the exact definitions of each class.

A second opinion from an experienced and trained orthodontist in CBCT diagnosis was sought when there were uncertainties regarding the classification. Through collaboration, a mutually agreed upon classification was established.

Subsequently, for a sample of 60 CBCT images selected randomly, an additional measurement was conducted two weeks later in order to assess the intra-rater reliability. The examiner’s classification was set as the benchmark for the following assessments by the three examiner groups.

A total of ten CBCT scans were selected at random, while ensuring that all defined stages were included at least once. These scans were assessed by three distinct groups of investigators, comprising dental students, orthodontic residents, and practicing orthodontists (each group comprised of five examiners). The assessment was conducted using a dynamic free-screening procedure. The examiners performed an independent evaluation and were required to orient the CBCT slices themselves, as instructed in training, to analyze midpalatal suture maturation. The number of examiners in each group was based on the study by Obuchowski, in which medical imaging studies were performed by 5–10 examiners [13]. The examiners were provided with training materials and an evaluation scheme in the form of a handout describing the radiological features at each maturity stage and accurate figures and flowcharts as described by Angelieri et al. to assess the midpalatal suture maturation stage. Prior to the assessment of the ten selected CBCT scans, all examiners were provided five different CBCT scans for training purposes. Examiners who had not acquired CBCT expertise were instructed using the software. Students were selected from higher semesters to ensure that they had adequate anatomical knowledge. A repeat assessment was performed after two weeks to measure the intra-rater reliability for each examiner. The software programs Medas (EDV Grundysteme, Margetshöchheim, Germany) and IBM SPSS Statistics 29 (IBM, Armonk, USA) were used for statistical analyses. Data distribution was analyzed using descriptive statistics and the Kolmogorov–Smirnov test. Weighted Cohen´s kappa was employed to determine the intrarater reliability for the benchmark classification and for inter- and intraexaminer agreement of the pooled performance for each group of dental students, orthodontic residents and orthodontists. Additionally, the agreement to the benchmark classification for each group was assessed using Cohen's kappa (k). The interpretation of kappa values was conducted in accordance with McHugh's classification system, which categorizes the level of agreement as follows: k ≤ 0.20 as none, k = 0.21 ≤ 0.39 as minimal, k = 0.40 ≤ 0.59 as weak, k = 0.60 ≤ 0.79 as moderate, k = 0.80 ≤ 0.90 as strong, and k > 0.90 as almost perfect [14].

For the level of intra-rater reliability exhibited by the individuals, a Kendall-tau correlation analysis was conducted and assessed in accordance with the classification system established by Chan, in which ratings of *r* < 0.3 were deemed poor, *r* = 0.3 ≤ 0.5 were considered fair, *r* = 0.6 ≤ 0.8 were categorized as moderately strong, and *r* > 0.8 were characterized as very strong correlation [15].

In addition to evaluate the deviation from the benchmark classification and compare the respective groups for diagnostic performance, stages A-E were coded in numbers 0–4. The classified values were summed for the respective examiners and the values of the benchmark classification were subtracted. Finally, the mean values for the respective groups were calculated, in analogy to the study of Barbosa et al. utilizing median values [16], and Wilcoxon pairwise comparison analysis was performed to compare for significant differences between the groups. The significance level for all statistical tests was set at *p* < 0.05.

## Results

### Intra-rater reliability for the benchmark classification

A strong intra-rater reliability (k = 0.85) for the benchmark classification, which involved classifying 179 CBCT scans and reclassifying 60 CBCT scans by one examiner, could be demonstrated.

### Intra-rater and inter-rater reliability for ten randomly chosen CBCTs for group performance

Student 1 showed poor and insignificant intra-rater reliability (tau = 0.2973, *p* = 0.23). Student 5 also showed insignificant but fair correlation (tau = 0.3244, *p* = 0.19), whereas student 2 showed a very strong correlation (tau = 1.000, *p* < 0.001). The other two students showed moderately strong correlation for intra-rater reliability (tau = 0.6494, 0.6844; *p* < 0.001).

For the pooled group of students the intra-rater reliability after a wash-out period of two weeks was to be considered as weak (k = 0.59).

Four orthodontic residents showed very strong intra-rater reliability (tau > 0.8, *p* < 0.001), of which one showed moderately strong intra-rater reliability (tau = 0.78, *p* < 0.05).

Intra-rater reliability according to weighted Cohen´s kappa for the group performance of orthodontic residents showed a strong level of agreement (k = 0.81) at the end of the wash-out period.

Three orthodontists showed very strong intra-rater reliability (tau > 0.8, *p* < 0.001) and two orthodontists showed moderately strong intra-rater reliability (0.7 < tau < 0.8, *p* < 0.05).

Weighted Cohen´s kappa analysis (k = 0.74) showed a moderate level of intra-rater agreement for the group after two weeks for orthodontists.

Inter-rater reliability of the three groups for the assessment of the ten CBCTs was to be considered as minimal (Table 1).

**Table 1:** Inter-rater reliability between the groups regarding diagnostic performance for ten randomly chosen CBCTs

*Examiners*	*k*
*Students vs. Orthodontic residents*	0.33
*Students vs. Orthodontists*	0.27
*Orthodontic residents vs. Orthodontists*	0.39

*k* weighted Cohen´s kappa

### Agreement level of the groups with regard to the benchmark classification

The degree of agreement between the benchmark classification and the actual classification is illustrated in Table 2. This table demonstrates a maximum deviation of three stages for all groups and the highest percentage of exact agreement for the orthodontists.

**Table 2:** Deviation of agreement with the benchmark classification of the three groups with different experience level

Agreement level	% Students	% Orthodontic residents	% Orthodontists	% Total
Exact **agreement**	40.0	60.0	72.0	57.3
**1 Stage disagreement**	32.0	16.0	20.0	22.7
**2 Stages disagreement**	18.0	10.0	0.0	9.3
**3 Stages disagreement**	10.0	4.0	8.0	10.7
**4 Stages disagreement**	0.0	0.0	0.0	0.0

**Fig. 1:**
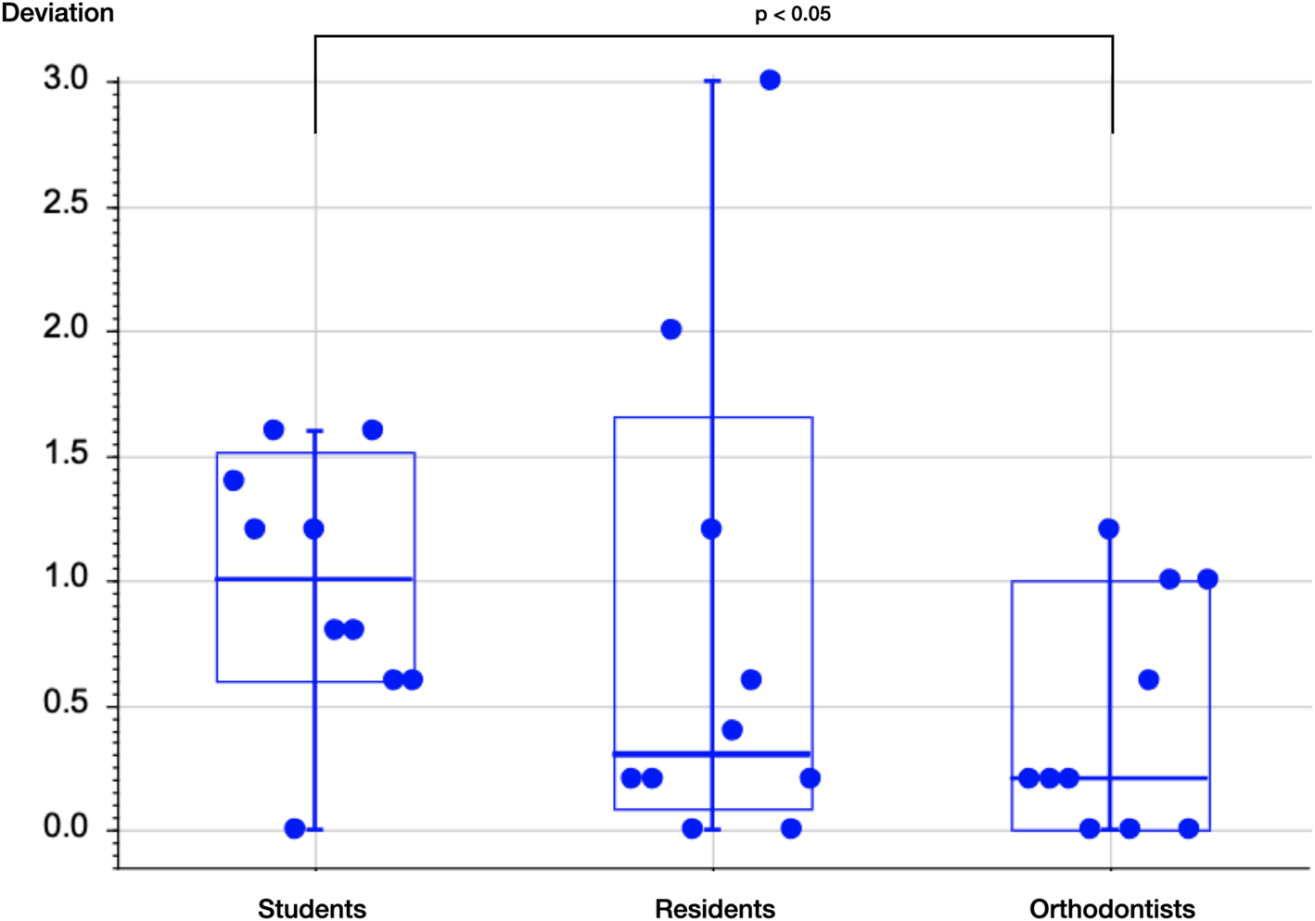
Boxplot diagram of the three examiner groups and their respective deviations from the benchmark classification; *p* = *p*-value

Comparison of the performance of the groups in terms of deviation from the benchmark classification and diagnostic precision, showed that the assessments performed by the orthodontists were significantly closer to the benchmark classification than those reported by the students (*p* < 0.05). However, no significant differences were observed in diagnostic performance between orthodontists and orthodontic residents and between orthodontic residents and students (*p* > 0.05) (Table 3, Fig. 1). This is supported by weighted Cohen´s kappa analysis for inter-rater reliability with regard to benchmark classification, which was minimal for the group of students (k = 0.34), and weak for orthodontic residents (k = 0.46) whereas orthodontists showed a moderate level of agreement (k = 0.68).

**Table 3:** Wilcoxon pairwise comparison of deviations from the benchmark classification

Pairwise group comparison	n	mean	SD	median	68%—CI		*p*
Students	10	0.980	0.512	1.000	0.600	1.514	0.24
Orthodontic residents	10	0.780	1.000	0.300	0.086	1.658	
Students	10	0.980	0.512	1.000	0.600	1.514	0.027^*^
Orthodontists	10	0.440	0.470	0.200	0.000	1.000	
Orthodontic residents	10	0.780	1.000	0.300	0.086	1.658	0.63
Orthodontists	10	0.440	0.470	0.200	0.000	1.000	

*n* number of patients, *SD* standard deviation, *p p*-value, *CI* confidence interval

^*^p-value < 0.05

## Discussion

Visualization and classification of the appearance of the midpalatal suture by CBCT images and its potential impact on treatment modality has been proposed by Angelieri et al. [5]. This proposal is not without controversy as resistance to maxillary expansion is influenced by multiple factors, which include not only intrinsic properties of the midpalatal suture but constraints imposed by surrounding structures [17]. Additionally no correlation could be found between ossification and the proposed classification stages, therefore the scientific rationale of the classification method has been questioned [18]. It has also been stated that the classification still lacks validity and is influenced by image quality and the examiner calibration, as interpretation of the midpalatal suture maturation stage is subjective [11]. This introduces potential variability in assessments among different examiners. The literature on the reproducibility of staging is inconclusive, and it has been suggested that future studies should include at least two examiners and a strict training protocol [8]. Our study compromises three different groups of examiners in terms of experience level, and each group consists of five examiners, and a training protocol was instituted.

The assessment for classification of midpalatal suture maturation in CBCT scans in our study had to be performed dynamically by scrolling to simulate a realistic clinical scenario, thereby enhancing the practical applicability of our findings. Barbosa et al. in contrast provided prepared axial slices of the midpalatal suture for the examiners as described by Angelieri et al. [5, 16]. This distinction is also important because examination of all midpalatal suture segments for assessment of the maxillary anatomy can be difficult when assessing a single slice.

The intra-rater reliability of the benchmark classification in this study was very strong. It can be inferred that the experience level of the benchmark examiner was likely high, considering 179 assessed CBCTs, which may have contributed to the high level of intra-rater agreement. A strong intra-examiner reliability for midpalatal suture classification using CBCT scans in adults was also reported by Angelieri et al. [19]. The use of multi-slice computed tomography (CT) scans to assess midpalatal suture maturation has shown to be reliable and reproducible [16], though it should be noted that our study utilized CBCT scans.

In this study, calibration of examiners was performed before evaluations in the three different groups, in accordance with the recommendations of a previous study [11]. However, unlike previous studies that used Cohen’s kappa values to evaluate intra-rater reliability [11, 16], Kendall’s tau correlation was additionally calculated in the present study. The use of Kendall ‘s tau correlation for assessment of intra-rater reliability has been reported in the literature [20], and this correlation analysis has been shown to be accurate for small datasets [21].

To our knowledge and on the basis of our review of the literature, only one study has compared the diagnostic performance of students and orthodontists for midpalatal suture classification using CBCT scans showing no significant difference regarding the experience level of the examiners. The results are restricted as the number of examiners were limited to one person per group [22]. Another study analyzed the performance of orthodontists and radiologists to classify the mipalatal suture maturation on CBCTs. Though it was stated they had varying level of experience, all were specialists in their field and thereby a high level of overall clinical experience could be assumed [16]. Our findings showed significant differences in the diagnostic performance of orthodontists and dental students, indicating that midpalatal suture maturation classification is related to the experience level of the examiner. These findings are in contrast to those obtained for volumetric cephalometric landmark identification, wherein inexperienced raters showed better performance than experienced raters in a comparison of dental students and orthodontic residents [23]. Nevertheless, significant differences between experienced practitioners and dental students have been reported for accurate measurement of the mandibular anatomy in CBCT scans [24]. These findings imply a major knowledge gap in the use of CBCT scans among dental professionals and indicate the need for adequate training [25].

In this study, none of the orthodontists showed almost perfect or no correlation for inter-rater reliability, but one dental student and one orthodontic resident showed almost perfect correlation. This may imply either very good conformity or very high bias owing to the previous classification. A notable aspect is that the classifications were performed unsupervised after calibrating the examiners. Nevertheless, since the median deviation from the benchmark classification for dental students was 1.0, in general, the error for students was approximately one classification stage, while it was lower for orthodontic residents and orthodontists. The group of orthodontists showed highest relative agreement of 92% and the least mean deviation with regard to the benchmark classification indicating that a high level of experience is necessary for the assessment of midplatal suture maturation stages. The inter-rater reliability was minimal between the groups of experience level and not higher than moderate with regard to benchmark classification indicating that the method is not free of subjectivity.

The study's prospective nature allows for meaningful comparisons between diverse examiner groups with varying levels of experience. The preliminary study is subject to certain limitations that must be taken into consideration. The within-group classification employs a relatively small sample size, which may affect the precision of the results. In order to assess the performance of midpalatal suture maturation, a subsample of 10 CBCT images was randomly selected from the original dataset. This approach may have resulted in certain stages being underrepresented, potentially impacting the generalizability of our findings. Therefore, to validate our findings, future studies with a more comprehensive sample that is representative of all stages would be beneficial. While we followed the ALARA (As Low As Reasonably Achievable) principle for radiation safety in our study, we acknowledge that the ALADAIP (As Low As Diagnostically Acceptable being Indication-oriented and Patient-specific) guideline would have been a more appropriate framework, especially for aligning radiation dose with the diagnostic necessity in pediatric imaging [26].

## Conclusions

The classification of midpalatal suture maturation on CBCT scans by visual inspection seems to be related to the experience level of the examiner. High level of experience seems favorable but does not necessarily ensure reliable assessment.

## Data Availability

All data are available on request by Annahita Arman (arman.05@hotmail.de).

## Abbreviations

CBCT: Cone-beam computed tomography
CT: Computed tomography
CVM: Cervical vertebral maturation
DICOM: Digital Imaging and Communications in Medicine
RME: Rapid maxillary expansion
SARPE: Surgically assisted rapid palatal expansion
MARPE: Miniscrew assisted rapid palatal expansion
